# Calf health from birth to weaning. III. housing and management of calf pneumonia

**DOI:** 10.1186/2046-0481-64-14

**Published:** 2011-10-21

**Authors:** Ingrid Lorenz, Bernadette Earley, John Gilmore, Ian Hogan, Emer Kennedy, Simon J More

**Affiliations:** 1 Herd Health and Animal Husbandry, UCD School of Veterinary Medicine, University College Dublin, Belfield, Dublin 4, Ireland; 2 Animal and Bioscience Research Department, Teagasc, Animal and Grassland Research and Innovation Centre, Grange, Dunsany, Co. Meath, Ireland; 3 Emlagh Lodge Veterinary Centre, Elphin, Co. Roscommon, Ireland; 4 Department of Agriculture, Fisheries and Food, Regional Veterinary Laboratory, Knockalisheen, Limerick, Ireland; 5 Animal and Grassland Research and Innovation Centre, Teagasc Moorepark, Fermoy, Co. Cork, Ireland; 6 Centre for Veterinary Epidemiology and Risk Analysis, UCD School of Veterinary Medicine, University College Dublin, Belfield, Dublin 4, Ireland

**Keywords:** Calf health, Disease prevention, Disease management, Suckler calf weaning, Castration, Dehorning, Housing, Pneumonia

## Abstract

Calfhood diseases have a major impact on the economic viability of cattle operations. A three part review series has been developed focusing on calf health from birth to weaning. In this paper, the last of the three part series, we review disease prevention and management with particular reference to pneumonia, focusing primarily on the pre-weaned calf. Pneumonia in recently weaned suckler calves is also considered, where the key risk factors are related to the time of weaning. Weaning of the suckler calf is often combined with additional stressors including a change in nutrition, environmental change, transport and painful husbandry procedures (castration, dehorning). The reduction of the cumulative effects of these multiple stressors around the time of weaning together with vaccination programmes (preconditioning) can reduce subsequent morbidity and mortality in the feedlot. In most studies, calves housed individually and calves housed outdoors with shelter, are associated with decreased risk of disease. Even though it poses greater management challenges, successful group housing of calves is possible. Special emphasis should be given to equal age groups and to keeping groups stable once they are formed. The management of pneumonia in calves is reliant on a sound understanding of aetiology, relevant risk factors, and of effective approaches to diagnosis and treatment. Early signs of pneumonia include increased respiratory rate and fever, followed by depression. The single most important factor determining the success of therapy in calves with pneumonia is early onset of treatment, and subsequent adequate duration of treatment. The efficacy and economical viability of vaccination against respiratory disease in calves remains unclear.

## Introduction

Calfhood diseases have a major impact on the economic viability of cattle operations, due to the direct costs of calf losses and treatment and the long-term effects on performance [1]. Furthermore, calf health was prioritised as one of the most important animal health issues facing the Irish livestock industry in a recent expert Policy Delphi study conducted on behalf of Animal Health Ireland (AHI [2]. As part of ongoing AHI work, a group of experts was commissioned to provide evidence-based advice on calf health and disease management to Irish farmers, agricultural advisers and veterinary practitioners. As an initial step, a review series on calf health from birth to weaning has been developed, specifically to provide a scientific evidence base to underpin advisory tools on calf health, and to identify gaps in current knowledge to be filled with targeted research. Even though the envisaged output will be specific for Irish husbandry systems, the scope of the reviews should make them useful for the same purpose elsewhere. The reviews cover both suckler and dairy calf management. However, due to the differences in the nature of these systems, some topics will deal mainly or exclusively with either dairy or suckler calves.

This paper is the last in a three part review series, which collectively focuses on calf health from birth to weaning. The first and second parts focus on general aspects of disease prevention [3] and the management of diarrhoea [4] in pre-weaned calves, respectively. In the current paper, we review housing and ventilation as well as prevention and management of pneumonia in recently weaned suckler calves and young dairy calves. There is a very distinct difference in the epidemiology of pneumonia of suckler calves and dairy calves. Most of the risk factors for pneumonia in young dairy calves are identical with what has been discussed in the first paper of this series [3], whereas additional risk factors for suckler calves will be discussed here.

### Housing and ventilation

#### Housing systems

Calves are born with functional thermoregulatory mechanisms. Therefore, healthy calves are readily able to deal with outdoor temperatures as long as they receive adequate amounts of energy and are provided with a dry, well-bedded and draft-free shelter [5]. The lower critical temperature, at which additional energy is needed for heat production, lies in the range of 10-15°C for calves in the first two weeks of life, declining with age to approximately 6-10°C in older calves, and is highly dependent on air speed [5,6]. The quality of bedding material is crucial for the amount of heat loss via conduction in calves lying down [6]. Deep straw bedding is superior to other bedding material in its efficacy as an insulator [6,7] and can provide a high 'nesting score' which has a preventive effect against calf respiratory disease in naturally ventilated calf barns [8].

Individual housing of dairy calves, either indoors or outside, is generally linked with improved calf health. There is long-term recognition of the benefit to dairy calf health of outdoor housing in hutches, especially for the prevention of diarrhoea and respiratory disease [9]. Hutches have been associated with lower morbidity and mortality in dairy calves [10-12]. No significant difference in either average daily gain, incidence of scours or pneumonia in the first two weeks of life was observed when comparing indoor and outdoor rearing in individual pens, whereby the indoor facilities have not been used for calf rearing before [13]. However, caring for calves in outdoor hutches can be uncomfortable in adverse weather conditions [10]. If calves are housed individually in naturally ventilated calf barns, solid dividers on the side of pens, together with a high 'nesting score', lower the risk for respiratory disease [8]. European legislation prohibits solid walls in individual calf pens and, while it allows calves to be kept individually for the first 8 weeks of life, it encourages group housing for animal welfare reasons (Council Directive 2008/119/EC).

There have been mixed reports on the impact of group housing of calves, both indoors and outside, on calf health. A number of authors have reported higher morbidity [14-19] and mortality [12] among group-housed pre-weaned dairy calves compared to individual housing. In contrast, two surveys report no difference in mortality between calves in group housing or individual housing [20,21]. In one out of two similar experiments Kung et al. found that fewer days of medication were needed in group-housed calves than in calves housed individually in hutches. However, the authors discuss the possibility that superior disease detection in individual housing could account for this finding [22]. Mortality was highest in larger calf groups (≥ 7 calves [23]) and tended to be lower in small groups compared with either individual housed calves or calves kept in large groups [19]. Calves in stable groups had significantly higher daily live weight gains than those in dynamic groups (where new calves were continuously introduced to and exited group housing). The prevalence of both diarrhoea and respiratory disease was more than twice as high among calves in dynamic compared to stable groups [24]. Grouping calves of similar age lowers the risk of respiratory disease compared to groups with wider age differences [25]. A higher incidence of respiratory disease but a lower incidence of diarrhoea has been identified when calves were reared in indoor groups compared with outdoor groups [26]. A review of group housing of dairy calves with different feeding systems concluded that group housing increases the risk of infection, especially in larger groups and thus requires more skills and poses more challenges to management [27].

In summary, there are a high number of experimental studies and surveys dealing in various ways with aspects of individual and group housing, as well as outdoor and indoor rearing, with sometimes contradicting results. Overall, outdoor individual hutches appear superior to indoor housing, and individual housing/small group housing appears superior to large group housing with regards to calf health.

#### Ventilation

Inadequate ventilation of calf barns increases the risk of disease due to a build up of high levels of humidity, noxious gases, dust and bacterial content [28]. Ammonia levels of less than 10 ppm are recommended [28], however, concentrations of 5 ppm already lead to adverse effects on the respiratory system in piglets [29]. Ammonia concentrations are enhanced by the accumulation of urine and faeces, which emphasises the need for regular cleaning and provision of dry bedding, together with adequate ventilation.

To prevent adverse conditions, at least 4 air changes per hour are needed in winter and up to 40 in summer [30]. Natural ventilation is achieved through wind and buoyancy in monopitch or duopitch houses, given that adequate air outlets (ridge opening: 5 cm width for every 3 m width of the building) and inlets (eave openings: at least half the space of ridge openings) [31], as well as sufficient difference in height between the openings is provided (not less than 1.5 m, but preferably 2.5 m) [6]. Recommended air space per calf is not less than 6 m^3 ^up to 6 weeks and 10 m^3 ^up to 12 weeks of age [6].

Problems can arise in naturally ventilated calf barns in cold and damp wintry conditions, when it can be impossible to keep relative humidity below an acceptable level of 85% [6]. Additionally, ventilation is often compromised by closing of air inlets in an attempt to prevent cold stress for the calves. If calves are housed in individual pens indoors, the barn climate often does not reflect the microclimate in the pens. Ventilation is impaired with an increasing numbers of solid panels surrounding the calf (solid walls in the back or front of the pen, top covers), leading to an increase in airborne microbes [8].

### Risk factors for disease in recently weaned suckler calves

#### Weaning management of the suckler calf

In suckler herds, calves generally remain with cows at pasture until they are weaned, usually between 5 and 9 months of age. In addition to removal from the cow, the weaning procedure may be compounded by other stressors occurring around the same time, e.g. change of diet (grass and milk to conserved feed with or without concentrates), change of environment (outdoors to indoors). Non-integrated systems often combine weaning with additional stressors such as transportation and marketing, prior to entry into feedlots [32]. Alterations in calf behaviour [33,34], hormonal mediators of stress [35,36] and consequently impaired immune function [37-41] are evident post-weaning. Furthermore, weaning is considered to be a predisposing factor to pneumonia in recently weaned suckler calves [42-44] and reducing the cumulative effects of multiple stressors around the time of weaning results in a less marked stress response. This strategy, combined with vaccination programmes and feeding concentrates pre-weaning (preconditioning), reduces subsequent morbidity and mortality in the feedlot [45] and provided the rationale for the 'Animal welfare, recording and breeding scheme for suckler herds' (Suckler Welfare Scheme) in Ireland [46].

Delaying housing of calves post-weaning reduces the magnitude of the stress response [41]. Also, calves supplemented with concentrate prior to weaning had a lesser reduction in some immune cells (i.e. γδ T lymphocytes), started consuming meal faster when housed indoors and spent more time lying down (rather than standing and walking) post-weaning compared with non-supplemented calves. Concentrate supplementation of suckler calves is often advocated as a means of reducing weaning stress in calves through familiarisation to a palatable feed [47] and has been reported to decrease morbidity in feedlots [48]. Management practices aiming at reducing stress at weaning include the use of anti-suckling devices (nose-clips) for a period prior to weaning [34,49,50], fence-line contact between calf and dam post-weaning [33,49,51,52] and a combination of both practices before complete separation [53]. The benefit of two-stage weaning with nose-clips and fence-line weaning on calf welfare has been questioned in a recent study, with no overall reduction of distress behaviours in weaned calves, but rather redistribution of these behaviours on the days post-attachment of nose-clip and on the days post-weaning [54].

#### Painful procedures

There are aspects of cattle production such as castration, disbudding and dehorning which cause pain and stress for the animal. Dehorning or disbudding of horned cattle is a mandatory requirement in many countries to reduce the risk of injuries to humans or other animals [55,56]. Cortisol and behavioural response to disbudding is significantly smaller than to amputation dehorning, implying that the latter is more painful [57]. Castration of male cattle is a routine procedure used in some husbandry systems to facilitate handling and modify carcass quality. It elicits physiological stress by increasing plasma cortisol concentrations, inflammatory reactions, pain associated behaviour, suppression of immune function and a reduction in performance [58-62]. Burdizzo castration causes a lower cortisol response than either surgical or rubber ring castration [63]. Physiological stress as measured by cortisol levels and inflammatory reactions (acute-phase proteins, scrotal swelling and surface skin temperature) are lower in 1.5 month old calves compared to older calves after Burdizzo castration [61].

Pharmacological methods (local anaesthesia, systemic analgesia using non-steroidal anti-inflammatory drugs, xylazine sedation) are available that are highly beneficial in alleviating the acute pain caused by castration, dehorning or disbudding [57,63]. National legislation regarding animal welfare and regulations concerning the usage of these drugs by veterinarians or producers vary between countries and have to be taken into account.

### Calf pneumonia

#### Aetiology and epidemiology

Pneumonia in pre-weaned calves is a multi-factorial disease involving a well known group of viruses (bovine herpesvirus 1, BoHV1; bovine respiratory syncytial virus, BRSV; parainfluenza 3 virus, PI3) and bacteria (*Mycoplasma bovis*, *Pasteurella multocida*, *Mannheimia haemolytica*, *Histophilus somni*), as well as calf-related and environmental risk factors. Bovine viral diarrhoea virus (BVDV) appears to play an important role, both in terms of immunosuppression and synergistic effects with other pathogens and also as a primary pneumopathogen [64]. Accumulating evidence has been found in recent years that bovine coronavirus plays a role in bovine respiratory disease [65].

Many aspects of respiratory disease in cattle have recently been reviewed [66], including issues specific to beef [67] and dairy [68] calves.

A number of factors are known to modify the risk of pneumonia in calf populations. Prior to weaning, single-suckled beef calves outdoors are at the lowest risk of pneumonia [67]. Outbreaks can occur due to sudden inclement weather [69]. However, if suckler calves are born and reared indoors, the incidence of pneumonia can be considerable [70]. In the period following weaning, pneumonia has a high prevalence in weaned beef calves. A large national survey of the US beef industry found that 14.4% of cattle placed in US feedlots acquire the disease [71]. The population wide-characteristics of weaned cattle of high respiratory disease risk include animals being of light weight, cattle from multiple origins, previous history of disease and cattle experiencing long journeys before arriving at a feedlot [72].

According to recent US data, respiratory disease is responsible for nearly a quarter of pre-weaned calf deaths and nearly half of weaned calf deaths in dairy replacement heifers [73]. Dairy calf rearing management and facilities vary widely between farms. Therefore, each of the previously mentioned risk factors at calf and environment level has to be considered when faced with an outbreak of calf pneumonia. Additional factors identified to increase pneumonia risk in housed calves include shared airspace with older animals, overcrowding, and power-washing of calf facilities while calves are still present [68].

#### Recognition

Cases of calf pneumonia may not be detected by the animal keeper, but are more likely to be missed than misdiagnosed, as Sivula et al. have shown that keeper diagnosis is only 56% sensitive but 100% specific [74].

Early signs of calf pneumonia include elevated respiratory rate, fever, serous nasal discharge and at the most mild depression or inappetence [75]. Since early treatment is the most important factor that prevents treatment failure, recognition at this stage would be preferable. The feasibility of daily measurement of the body temperature in high-risk periods is highly dependent on the housing system and handling facilities. When treatment is based on rectal temperature, thresholds of 40-40.3°C for feedlot cattle and 39.7°C for calves have been suggested [28]. If measurement of the body temperature is not practical, early recognition and the success of treatment relies on good observational skills of the animal keeper. Evaluating calves for treatment using a screening system, such as the calf respiratory scoring chart developed at the University of Wisconsin, which is based on rectal temperature, character of nasal discharge, eye or ear appearance and presence of coughing, has been recommended for dairy calves [68,76]. Apley [77] suggests that treatment should be instituted on recognition of depression with undifferentiated fever, with depression being the more important of these two parameters.

#### Diagnostic tests

The value of diagnostic tests in calf pneumonia is somewhat limited due to the multifactorial nature of the disease and the uncertainty if the pathogens recovered from samples are causative to the disease [78]. Most outbreaks can be successfully managed using the principles for treatment described above, and diagnostic tests cannot replace the examination of management practices and facility design in cases of recurrent outbreaks [28].

If animals are selected for ante-mortem sampling they should be in the early stages of the pneumonic process, before treatment, and should show typical signs of the process affecting the group [79]. Nasal swabs should only be used to identify upper respiratory tract viruses [79]. Deep nasopharyngeal swabs, positive for *M. haemolytica *and *M. bovis*, have been demonstrated to be representative of isolates present in the lungs [80]. Samples obtained from transtracheal wash and/or bronchoalveolar lavage (BAL) can be used for virology, bacteriology, cytology and parasitology [79]. However, the presence of bacterial isolates in nasopharyngeal swabs or tracheal and/or bronchoalveolar lavage needs to be interpreted with caution, in light of recent studies in which 63% of healthy calves were culture positive for bovine bacterial pathogens from BAL fluid [78]. In one study, a high level of pathogens was found in the lungs of calves on arrival at feedlots [81]. However, in another, the lungs were virtually sterile at the time of slaughter [82]. Bacteria may be more likely to frequent the lung during high stress periods due to impairment of the mucociliary escalator mechanism [81].

Postmortem examination of untreated animals in the early stages of calf pneumonia can be useful, whereas repeatedly treated animals with chronic pneumonia are usually of little diagnostic value [79]. Faeces should be examined for lungworm larvae, even though false negatives may occur if the animals are sampled before adult lungworm become patent [83].

#### Treatment

Antibiotic treatment of bacterial pneumonia must be sufficient in duration and, most crucially, early enough to prevent lesions forming that may resist both therapy and regeneration of normal lung parenchyma [28]. The emphasis should be on early treatment and first treatment success in cases of calf pneumonia since the outcome for those animals that fail to respond successfully to first treatment is poor. Typically, one third to two thirds of animals that do not respond to initial therapy are permanently affected or lost [84].

The effectiveness of metaphylaxis (defined as mass medication of all animals on arrival in the feedlot) in reducing morbidity rates associated with pneumonia in feedlots is variable [72]. A recent meta-analysis of North American studies estimated a decrease in mortality and morbidity of 2% and 26%, respectively for animals that received antimicrobial treatment on arrival in the feedlot. The average daily weight gain was 0.11 kg higher in these animals in comparison with calves not receiving metaphylactic treatment [85].

The use of antimicrobials for prevention (prophylaxis or metaphylaxis) of calf pneumonia has to be seen in the context of increasing pressure on the veterinary profession to promote prudent use of antibiotics, noting that indiscriminate use of antibiotics promotes the selection and subsequent proliferation of antibiotic-resistant strains of bacteria [86]. The European Parliament recently called for a review of current practices of prophylactic use of antimicrobials [87].

Non-steroidal anti-inflammatory drugs (NSAIDs) have shown to reduce pyrexia [88-91], clinical signs [89,90], and lung pathology [90-92], and improve average daily weight gains [92] in calves with respiratory disease compared to untreated calves or calves only treated with antimicrobials. Other studies, however, have not found significant differences between treatment groups [93,94]. The cost-efficiency of additional anti-inflammatory therapy in bovine respiratory disease is uncertain [28].

It has been suggested that pneumonic animals should be isolated in appropriate facilities [42]. However, there is little experimental evidence to quantify the benefits and it may lead to practical difficulties.

#### Specific prevention (vaccination)

The efficacy and economical viability of vaccination against respiratory disease in calves remains uncertain. Although substantial relevant literature is available, a consensus, underpinned by robust scientific findings, has not yet been achieved. The evaluation of vaccine efficacy, and the interpretation of trial results, is complicated by the nature of bovine respiratory disease, and in particular the multitude of pathogens and environmental stressors that contribute to disease development. Additionally, the disease pattern of pneumonia in calves can vary under a variety of husbandry systems, as a consequence of differing challenges at different points in the rearing period.

Modified live vaccines, inactivated vaccines, or subunit vaccines are available for most of the major pathogens. For the most part, relevant research has focused on the ability of these vaccines to trigger an immune response or to decrease clinical disease and pathogen shedding in challenge trials. Although field trials are needed to provide conclusive evidence of vaccine efficacy under field conditions [28], few of these have been reported. In a very recent paper, where a field trial was conducted, Windeyer [95] could find no beneficial effect of vaccination with a multivalent respiratory vaccine in a large population of young dairy calves with a low incidence of failure of passive transfer.

Substantial data are available on pneumonia in feedlot cattle, predominantly from the USA. These data provide useful insights, albeit for different husbandry systems, of the impact of multiple stressors on recently weaned suckler calves. The feedlot studies clearly indicate that vaccination provides best results when carried out in healthy animals and prior to the exposure to defined stressful events, such as weaning, marketing, transportation and associated changes in environment. Vaccination together with further management measures (preconditioning) in suckler calves before weaning has been shown to be beneficial on the performance of these animals after arrival in feedlots. However, in these studies the benefit of vaccination and management procedures cannot be assessed independently [45]. Perino and Hunsaker [96] conducted a review of field trials to assess the efficacy of mono- or multivalent vaccination against bovine respiratory disease without preconditioning. Using outcomes of morbidity and mortality in feedlot cattle, these authors identified 9 studies with positive and 13 studies with neutral or negative outcomes.

Early studies with formalin-inactivated experimental **Bovine Respiratory Syncytial Virus **vaccines demonstrated enhancement in disease severity after subsequent infection [97]. Meanwhile the safety and efficacy of modified live and inactivated BRSV vaccines have been demonstrated in experimental challenge trials, whereby reduced clinical disease and lung lesions have been found in most [98-100] but not all [101] trials. Monovalent BRSV vaccines have rarely been tested in field trials. Van Donkersgoed et al [102] tested a modified live BRSV vaccine in eight different scenarios in calves and yearlings and found only significant reduction of cases of bovine respiratory disease in 2 groups. However, all animals were comingled throughout the trials, which suggests that the development of herd immunity could have affected the apparent efficacy of the vaccine.

Young calves do not produce specific antibodies after vaccination in the presence of maternally derived immunity. For this reason, it is commonly believed that maternal antibodies can interfere with the efficacy of vaccination [103]. To overcome this issue, research in recent years has focused on the use of the mucosal immune system for vaccination. Intranasal vaccination against BRSV proved effective in challenge studies in calves without maternal antibodies [104-107]. In calves with maternal antibodies against BRSV, protective effect of intranasal vaccination compared to an unvaccinated control group was confirmed in two studies [106,108]. However, Ellis et al. [105] could not prevent clinical disease and lung lesions in calves with or without maternal antibodies that were challenged 4.5 months after intranasal vaccination with a modified-live virus vaccine.

Depending on virus virulence and host resistance, **Bovine Herpes Virus 1 **infection can cause clinical pictures from severe classical Infectious Bovine Rhinotracheitis (IBR) to no clinical signs at all. Regardless, infection will lead to latency in the host. In relation to calf pneumonia, it is important to consider the immunosuppressive effect of BoHV1 infection as well as BoHV1 reactivation [109]. Conventional BoHV-1 vaccines containing modified live virus are very effective, inducing both humoral and cellular immune responses[110]. However, they can establish latency and can be reactivated with adverse effects on pregnant cows or young calves in contact with the vaccinated animals. Inactivated vaccines, on the other hand, are safe but less efficacious as they only stimulate humoral immunity [109]. Gene-deleted (gE^-^) modified live virus and inactivated marker vaccines, to distinguish vaccination from field virus infection, have been developed and are commercially available [111]. Vaccination of newborn calves, especially in the presence of maternal antibodies, poses the same challenges as described for BRSV vaccination and is in the focus of current research [111]. Intranasal vaccination of seronegative newborn calves decreases clinical signs in a challenge trial [107]. However, the situation is complicated by the potential for establishment of seronegative latent carriers in calves with specific maternal antibodies through field virus infection as well as through vaccination with modified-live vaccines [112,113]. In Europe, the use of conventional vaccines is prohibited in some countries. Further, in countries with existing eradication programmes, regulations regarding vaccination need to be considered [114].

Vaccines against the major bacterial pathogens involved in bovine respiratory disease (***Pasteurella multocida*, *Mannheimia haemolytica*, *Histophilus somni***) can decrease clinical signs in challenge models but are rarely tested in monovalent form in field trials [115]. Aubry et al. [116] were unable to identify any decrease in treatments for respiratory disease in young dairy calves vaccinated with a modified-live *Mannheimia haemolytica *and *Pasteurella multocida *vaccine.

## Conclusions

Pneumonia is a significant cause of morbidity and mortality in calves, both during the pre-weaning period and shortly following weaning. A range of events are linked with increased disease risk, including weaning management, painful procedures, housing systems and ventilation and effective preventive measures have been demonstrated. The management of pneumonia in calves is reliant on a sound understanding of aetiology and of relevant risk factors and of effective approaches to diagnosis and treatment.

## Conflict of interest statement

None of the authors of this paper has a financial or personal relationship with other people or organisations that could inappropriately influence or bias the content of the paper. The Technical Working Group includes employees of Pfizer Inc. (CC) and Volac Ireland (LG); these companies played no role in the design, development or journal submission of this review series.

## Authors' contributions

IL, BE, JG, IH and EK drafted the manuscript and compiled the literature. All authors made substantial inputs to the review, critically discussed the progressing manuscript and approved the final manuscript.
